# The influence of Multiwave Locked System (MLS) laser therapy on clinical features, microcirculatory abnormalities and selected modulators of angiogenesis in patients with Raynaud’s phenomenon

**DOI:** 10.1007/s10067-014-2637-8

**Published:** 2014-05-13

**Authors:** Anna Kuryliszyn-Moskal, Jacek Kita, Agnieszka Dakowicz, Sylwia Chwieśko-Minarowska, Diana Moskal, Bożena Kosztyła-Hojna, Ewa Jabłońska, Piotr Adrian Klimiuk

**Affiliations:** 1 Department of Rehabilitation, Medical University of Bialystok, M. Sklodowskiej-Curie 24A, 15-276 Bialystok, Poland; 2 Department of Clinical Phonoaudiology and Logopedics, Medical University of Bialystok, Bialystok, Poland; 3 Department of Immunology, Medical University of Bialystok, Bialystok, Poland; 4 Department of Rheumatology and Internal Medicine, Medical University of Bialystok, Bialystok, Poland

**Keywords:** Angiogenesis modulators, Multiwave Locked System (MLS) laser therapy, Nailfold videocapillaroscopy (NVC), Raynaud’s phenomenon

## Abstract

The aim of this study was to investigate the influence of the Multiwave Locked System (MLS) laser therapy on clinical features, microvascular changes in nailfold videocapillaroscopy (NVC) and circulating modulators releasing as a consequence of vascular endothelium injury such as vascular endothelial growth factor (VEGF) and angiopoietin 2 (Ang-2) in patients with primary and secondary Raynaud’s phenomenon. Seventy-eight RP patients and 30 healthy volunteers were recruited into the study. All patients with RP received MLS laser irradiation for 3 weeks. Clinical, NVC and laboratory investigations were performed before and after the MLS laser therapy. The serum concentration of VEGF and Ang-2 were determined by an enzyme-linked immunosorbent assay (ELISA). After 3 weeks of MLS laser therapy, the clinical improvement manifested by decreasing of the number of RP attacks, mean duration of Raynaud’s attack and pain intensity in RP patients was observed. After MLS laser therapy in 65 % of patients with primary and in 35 % with secondary RP, an increase in the loop number and/or a reduction in avascular areas in NVC were observed. In comparison with a control group, higher serum concentration of VEGF and Ang-2 in RP patients was demonstrated. After MLS laser therapy, a reduction of Ang-2 in both groups of RP patients was found. Our results suggest that NVC may reflect microvascular changes associated with clinical improvement after MLS laser therapy in patients with primary and secondary RP. Ang-2 serum levels may be a useful marker of microvascular abnormalities in RP patients treated with MLS laser therapy.

## Introduction

Raynaud’s phenomenon (RP) is a vascular disorder characterized by recurrent vasospastic response of the fingers and toes to cold or emotional stimuli. It can be primary (idiopathic) or secondary (Raynaud’s syndrome) related to a number of different pathological conditions, including rheumatic diseases [1].

Although the pathogenesis of RP is poorly understood, current data points to three main pathophysiological mechanisms: vascular abnormalities, disturbances in the neural control of vascular tone and intravascular factors including circulating mediators and they influence on the balance between vasoconstriction and vasodilatation [2]. Angiogenic factors such as vascular endothelial growth factor (VEGF) and angiopoietin-2 (Ang-2) are the main regulators of angiogenesis, playing an essential role in vascular remodelling [3, 4]. Ang-2 as an autocrine mediator of the endothelium makes the vessel hypersensitive to the effects of pro-inflammatory cytokines and VEGF, resulting in vessel destabilization [5, 6]. Furthermore, it has been shown that VEGF and Ang-2 reflected the dysregulation of endothelium, leading to the development of the main clinical manifestations in patients with systemic sclerosis [7].

RP is a vascular disorder that involves digital arteries, pre-capillary arterioles and subcutaneous arteriovenous shunts, leading to the microvascular abnormalities with a broad spectrum of clinical manifestations. According to recent studies, microvascular abnormalities, manifested as RP, can be a predictor of severe underlying disease with reduced life expectancy [1]. Nailfold videocapillaroscopy (NVC) has been proposed as a first-line investigation in the early differential diagnosis of RP and connective tissue diseases [8–10]. In our previous studies, we demonstrated the usefulness of NVC as a non-invasive method for the evaluation of microvascular involvement in patients with systemic lupus erythematosus (SLE), rheumatoid arthritis (RA) and other connective tissue diseases (CTD) [11, 12].

Although new approaches to the pathogenesis and early diagnosis of RP are continually improving, still no universal guidelines for the treatment of RP exist. Treatment for RP depends on its severity and for mild disease is generally conservative [12]. The results of standard treatment for RP showed a weak improvement due to side effects and poor compliance [13, 14]. Therefore, searching for non-pharmacologic treatment methods is the focus of many recent studies [15].

Laser therapy in low doses has a beneficial effect in patients with primary and secondary RP [16]. It has been demonstrated that low-level laser therapy reduces significantly the frequency and severity of vasospastic attacks, but the mechanism of action and the degree of improvement of laser biostimulation in RP patients remain unclear [17].

Multiwave Locked System (MLS) laser therapy, characterized by a synchronized emissions of two wavelengths of 808 nm (in continuous mode) and 905 nm (as a pulsed laser light), is a new technique used in order to increase the effect of laser irradiation [18]. Two emissions are absorbed by different mitochondrial complexes and can affect cellular energy metabolism by acting on multiple sites in the cellular respiratory chain at the same time. Continuous emission is absorbed by the cytochrome oxidase which activation promotes the production of ATP, leading to the anti-inflammatory and anti-oedematous effects by stimulating microcirculation and influencing on the synthesis and degradation of inflammatory mediators [19]. Pulsed emission reduces pain through an effect on the superficial nociceptors and afferent nervous fibres, influencing on the nerve conduction [20]. The result of this emission is an increase of the nociceptive threshold and in a consequence—a reduction of pain sensation. Synchronization of both radiation components intensifies the analgesic, anti-inflammatory and anti-oedematous effect, increasing the intensity of the therapeutic effect on both pain and inflammation [19, 21]. Therefore, it is possible that MLS radiation can interact with deep located tissue and influence on the permeability of the cellular membrane, vessel walls (anti-inflammatory and anti-edematous effect) and peripheral nervous system (analgesic effect).

Despite the growing interest in MLS laser therapy as a new treatment method, the mechanisms underlying the effect on microcirculation in patients with RP are unclear. Therefore, the aim of this study was to investigate the influence of MLS laser therapy on clinical features, microvascular changes in nailfold videocapillaroscopy and circulating modulators releasing as a consequence of vascular endothelium injury such as VEGF and Ang-2 in patients with primary and secondary Raynaud’s phenomenon.

According to our knowledge, the present study is the first analysis of the influence of MLS laser therapy on microvascular abnormalities and modulators of angiogenesis in patients with Raynaud’s phenomenon.

## Subjects and methods

### Patients

Seventy-eight outpatients with RP (75 women and 3 men; mean age 42.8 years, range 19–77) of the Department of Rehabilitation of the Medical University of Bialystok, Bialystok, Poland, were recruited into the study. All RP patients were classified into two groups, those with primary RP, 38 cases (including 36 women and 2 men; mean age 36.5 years, range 19–77), fulfilling the criteria of LeRoy and Medsger [22], exhibited exclusively Raynaud’s phenomenon without any clinical or laboratory signs of the presence of a systemic autoimmune disease and 40 patients with secondary RP (39 women and 1 man; mean age 48.9 years, range 26–66). Patients with primary RP had absence of NVC scleroderma pattern and negative findings of autoantibody assays [22]. The group of secondary RP included 28 patients with systemic sclerosis (SSc), six patients with systemic lupus erythematosus (SLE) and six cases with undifferentiated connective tissue disease (UCTD), according to the international criteria [23–26]. Patients with the history of previous contact with vasodilators or immunosuppressive therapy with cyclosporine or cyclophosphamide, which could influence on the microvasculature, were excluded [27]. The characteristics of the patients’ groups are shown in Table 1.

**Table 1:** Clinical and capillaroscopic differences in patients with primary and secondary Raynaud’s phenomenon (RP) before MLS laser treatment

Characteristics	RP together (*n* = 78)	Primary RP (*n* = 38)	Secondary RP (*n* = 40)	*p* value
Sex (M/F)	3/75	2/36	1/39	NS
Age (years) (median, range)	43.5 (19–77)	29.5 (19–77)	53.0 (26–66)	NS
Disease duration (years, range)	9.0 (1–40)	6.0 (2–40)	12.0 (1–30)	NS
Number of RP attacks per week (median, range)	14.0 (1–76)	6.0 (1–76)	20.0 (1–75)	<0.02
Mean duration of RP attack (minutes) (median, range)	15.0 (5–120)	15.0 (10–120)	15.0 (5–60)	NS
VAS (mm) (median, range)	34.0 (0–96)	19.0 (0–96)	46.0 (0–87)	NS
Capillaroscopy score				
Score 0 (*n*/%)	21/26.9	21/55.3	0	<0.001
Score 1 (*n*/%)	19/24.4	17/44.7	2/5.0	<0.01
Score 2 (*n*%)	15/19.2	0	15/37.5	<0.01
Score 3 (*n*/%)	23/29.5	0	23/57.5	<0.001
ANA (*n*/%)	29/37.2	0	29/72.5	<0.001

Patients had not been treated with drugs which have effects on vascular remodelling and immunosuppressive agents. Patients were treated, when required, with diuretics (eight persons), anti-malarial drugs (six patients) and no more than 5 mg/day prednisone equivalent (seven patients). The control group consisted of 30 healthy volunteers, matched for sex and age, with no signs of RP.

The Ethical Committee of the Medical University of Bialystok, Bialystok, Poland, approved the study, and informed consent was obtained from all participants before entry to the examination.

### Clinical and laboratory analysis

Clinical, capillaroscopy and laboratory investigations were performed on the days of blood sample collection, i.e. 1 week before the start of treatment and 1 week after the last session (4 weeks after the first session) of the MLS laser therapy. The data were collected only during the winter months.

One week before the start of treatment, during the 3 weeks of the MLS laser therapy, and for 1 week after the end of exposure sessions, patients were instructed to record any Raynaud’s attacks in diaries. Number of attacks, the mean duration of Raynaud’s attack and severity of pain, assessed by means of a 10-cm visual analogue scale (VAS), had to be recorded daily at bed time.

Serum samples were frozen at −80 °C immediately after collection. Examinations included pulmonary and renal function tests, chest X-ray, renal sonography as well as erythrocyte sedimentation rate (ESR) and total antinuclear antibodies (ANA). ANA were detected by indirect immunofluorescence on human Hep-2 cell substrate (Viro-Immun Labor Diagnostica GmbH, Germany) and considered as positive when a dilution higher than that of 1:80 was obtained. The serum concentration of VEGF and Ang-2 were determined using an ELISA kit (Quantikine, R&D Systems). Assays were performed strictly according to the manufacturers’ instructions.

### NVC

NVC was performed in all patients and healthy volunteers using a stereomicroscope SZ 4045 with a final magnification of ×200 (Olympus, Germany). A fibre optic light source and filter provided cold illumination. The optical microscope was connected to a colour digital camera and a personal computer with a high-resolution colour monitor (14 in.) (Olympus, Germany). Four consecutive fields extending over 1 mm in the middle of the nailfold were studied per finger using image analysis software (Imaging Software Cell*, Olympus Soft Imaging Solutions). The images taken at the time of examination were analyzed by the same experienced investigator (AKM) without knowledge of the patient’s clinical diagnosis. The investigator was blinded also referring to the capillary images recorded as a pre- and post-treatment with MLS laser therapy.

Each patient was acclimatized for 20 min at room temperature of 20–24 °C prior to the examination. The nailfolds of all fingers except the thumbs and fingers affected by recent local trauma were examined in each patient. To obtain the best visibility of the microvasculature, a drop of immersion oil was placed on the nailfold bed. Only the capillaries in the distal row of the nailfold were analyzed.

The intensity of the morphological changes was evaluated on the basis of semiquantitative method which included the parameters: loop density; capillary length variability; percent of loops with architectural derangement such as tortuous, meandering, enlarged/giant, ramified or bushy capillaries; irregular distribution of the capillary array; and the presence of extravasations into perivascular tissue. All parameters were defined on the basis of previous classifications [9, 11] as follows: dilated capillaries, microvessels with a diameter of the arterial limb wider than 0.015 mm (=15 μm) or a venous limb wider than 0.020 mm (=20 μm); giant capillary loops, homogenously enlarged loops with a diameter wider than 0.050 mm (=50 μm); elongated capillaries, microvessels with length greater than 0.500 mm (=500 μm); microhemorrhage, presence of a dark red mass characterized by hemosiderin deposit derived from capillary injury; tortuosity, a variation of the typical harpin capillary shape; and avascular area, a distance between two adjacent capillary loops longer than 0.500 mm (=500 μm). The capillaroscopic findings were graded according to previous studies [28–30] with modifications ranging from 0 to 3 where score 0 indicated normal feature (>8 capillaries/mm, typical hairpin-shaped loops arranged in parallel rows); 1, mild (6–8 capillaries/mm, <33 % changed capillary loops with non-homogenous distribution or size of loops, elongation of the loop or shortened loops and the absence of haemorrhages); 2, moderate (33–66 % morphologically changed capillaries, enlarged loops, diminished loop density and microhaemorrhages); and 3, severe changes (more than 66 % dilated capillaries with heterogeneous features of angiogenesis with a variable capillaroscopic pattern, avascular areas and microhaemorrhages). The mean score for each subject was obtained from the analysis of all fingers.

### MLS laser therapy

All patients with RP received two-wave MLS laser (ASA Company) irradiation (808 and 905 nm) 5 days per week during the period of 3 weeks, with the following parameters: power 3.3 W (3,300 mW), frequency 1,500 Hz, energy 129.3 J, energy density 1.6 J/cm^2^ and procedure time 2.5 min on single hand. The laser scanner covered the fingertips and the dorsal parts of both hands.

### Statistical analysis

The significance of differences between control group and particular patient groups were tested using Mann–Whitney rank sum test. Wilcoxon signed rank test was used to evaluate the differences before and after MLS laser therapy. The probability of differences in frequency distributions was determined by the chi-square test or Fisher’s exact test. The data were correlated by Spearman rank order correlation. *p* values lower than 0.05 were considered as statistically significant.

## Results

The characteristics of 78 patients with primary and secondary RP are shown in Table 1. Before the MLS laser therapy, no significant differences in age, sex, the duration of symptoms and the pain intensity assessed by visual analogue scale (VAS) between two groups of patients with primary and secondary RP were seen. The mean duration of RP attack in patients with primary RP was longer, but not significantly, than in the group with secondary RP (29.6 vs 22.1 min) (Table 1).

After the MLS laser therapy, the number of RP attacks, their duration and intensity measured by VAS scale was significantly reduced in the both groups of RP patients (*p* < 0.001) (Table 2).

**Table 2:** Clinical characteristics of patients with primary and secondary Raynaud’s phenomenon (RP) before and after MLS laser therapy

		Primary RP	*p* value	Secondary RP	*p* value
Number of RP attacks per week (median, range)	Before MLS laser therapy	6.0 (1–76)	<0.001	20.0 (1–75)	<0.001
	After MLS laser therapy	5.0 (0–35)		15.0 (0–70)	
Mean duration of RP attack (min) (median, range)	Before MLS laser therapy	15.0 (10–120)	<0.001	15.0 (5–60)	<0.001
	After MLS laser therapy	12.5 (0–60)		10.0 (0–60)	
VAS (mm) (median, range)	Before MLS therapy	19.0 (0–96)	<0.001	46.0 (0–87)	<0.001
	After MLS laser therapy	14.5 (0–68)		31.0 (0–71)	

NVC patterns of 78 RP patients and 30 healthy volunteers were examined in this study 1 week before the start of treatment and 1 week after the MLS laser therapy. Before treatment in 21 of 38 (55.3 %) patients with primary RP and in 17 of 38 (44.7 %) persons, normal capillaroscopic pattern and minor abnormalities (score = 1) were found, respectively. All patients with secondary RP showed abnormalities in capillaroscopy. In this group, major capillary abnormalities (score = 3) were observed in 23 of 40 (57.5 %) patients, moderate changes (score = 2) were present in 15 of 40 (37.5 %) and, finally, 2 of 40 (5 %) persons had minor abnormalities (Table 1).

The capillaroscopic feature in the control group showed hairpin capillaries in a parallel arrangement. In isolated tortuous capillary loops and meandering, elongated capillaries were found in four persons (13.3 %) of the healthy volunteers. ANA were detected in 29 patients and were present only in the group with secondary RP (72.5 %).

After the MLS laser therapy, the number of patients with primary RP and normal capillaroscopic patterns increased from 21 (55.3 %) to 29 (73.7 %) persons. Furthermore, in patients with secondary RP, a reduction of the number of patients with severe capillaroscopic abnormalities from 23 (57.5 %) to 17 (42.5 %) cases was observed. Moreover, in seven from 15 patients with moderate changes (score = 2), an increase of capillaroscopic images with regular distribution and normal capillary density was found. After the MLS laser therapy in 14 of 40 (35 %) patients with secondary RP, an increase in the loop number and a reduction in avascular areas, capillary alterations and microhaemorrhages in capillaroscopy were observed (data not shown).

**Fig. 1:**
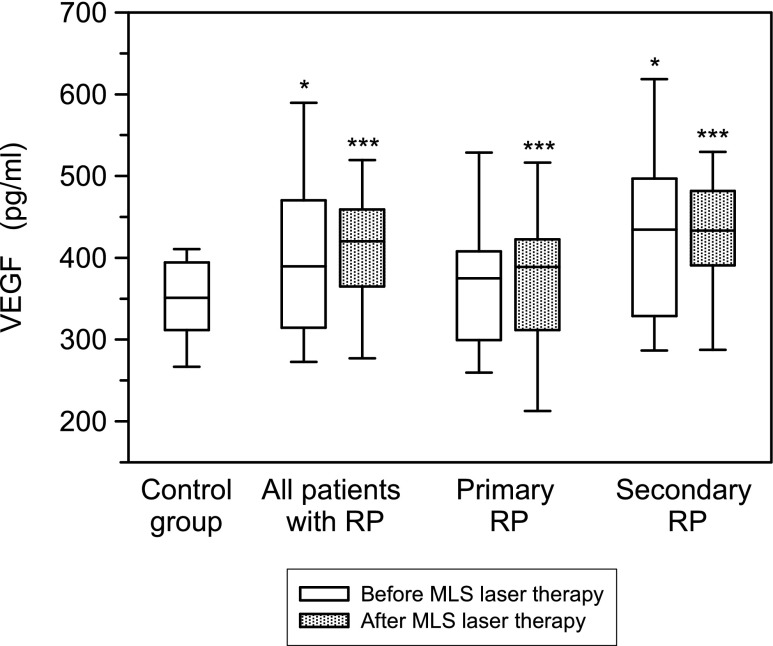
Serum concentrations of vascular endothelial growth factor (VEGF) in RP patients before and after MLS laser therapy assessed by ELISA technique. *Box plots* represent median (*line*), 25th and 75th percentiles (*box*) and 10th and 90th percentiles (*whiskers*). Significance of differences between control group and particular subgroups of patients were expressed as *p < 0.05, ***p < 0.001

**Fig. 2:**
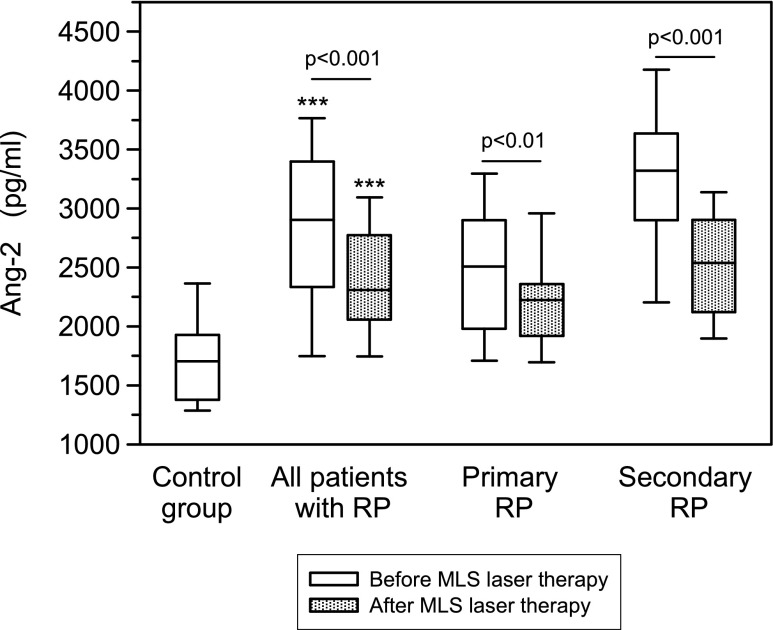
Serum concentrations of angiopoietin 2 (Ang-2) determined and presented as described in the legend of Fig. 1

Serum levels of VEGF and Ang-2 concentrations were determined in 78 RP patients, including 38 patients with primary RP and 40 cases with secondary RP, and in 30 healthy volunteers. The RP patients had significantly elevated concentrations of VEGF and Ang-2 compared with age-matched healthy volunteers (*p* < 0.05, *p* < 0.001, respectively). Before the MLS laser therapy, the serum concentrations of VEGF (*p* < 0.05) and Ang-2 (*p* < 0.001) were significantly higher in patients with secondary RP in comparison to the group with primary RP (data not shown). After the MLS laser therapy, serum Ang-2 levels decreased statistically significant in patients with primary and secondary RP compared with the values before the treatment. In contrary, no significant differences of VEGF serum concentrations between the RP patients before and after MLS laser therapy were found (Figs. 1 and 2).

Before the therapy, RP patients with severe and moderate changes in nailfold capillaroscopy (score >1) showed higher VEGF and Ang-2 serum levels than persons without or with mild changes; however, no significant differences were observed (data not shown). After the MLS laser therapy, serum levels of Ang-2 were significantly lower than before the treatment in all RP groups according to capillaroscopic score (*p* < 0.05 according to scores 0, 1 and 2 and *p* < 0.001 in patients with score = 3) (data not shown).

In patients with primary RP, significant positive correlation between duration of attack and the intensity of pain (according to VAS scale) was observed (*r* = 0.65, *p* < 0.001). Before the treatment, a significant positive correlation between the microvascular changes in NVC and the number of RP attacks (*r* = 0.24, *p* < 0.05) as well as the intensity of pain measured by VAS scale (*r* = 0.27, *p* < 0.02) in patients with primary and secondary RP was found. After the MLS laser therapy, the severity of capillaroscopic abnormalities correlated significantly with the intensity of RP attack (*r* = 0.23, *p* < 0.05). Moreover, Ang-2 serum level was positively correlated to the duration of RP (*r* = 0.42, *p* < 0.01) and negatively to the duration of RP attack (*r* = −0.34, *p* < 0.05) (data not shown).

## Discussion

Although RP can be manifested clinically as an isolated, acute vascular disorder with a benign course, it may precede the future development of systemic connective tissue diseases leading to serious complications associated with reduced quality of life and shortened life expectancy [28, 31–33]. Proposed therapeutic interventions in RP include a broad spectrum of therapies, from pharmacological treatment to surgery, reserved for complicated conditions [13, 14]. The unsuccessful effects and limitations of the pharmacological treatment are the reason of searching for new therapeutical methods, such as non-pharmacologic therapy [15, 16]. One of these methods is new a technique of laser irradiation—MLS laser therapy consisted of synchronized generation of two-wave laser radiation leading to analgesic, anti-inflammatory and anti-oedema effects [18, 19]. However, no studies exist on the relationship between the clinical effectiveness of MLS laser therapy, microvascular abnormalities in NVC and immunological parameters of endothelial cell damage in patients with primary and secondary RP.

In the present study, after 3 weeks of MLS laser therapy, the clinical improvement manifested by decreasing of the number of RP attacks, duration of Raynaud’s attack and pain intensity measured by VAS scale in both groups of RP patients was observed. The effectiveness of MLS laser therapy has also been observed in knee osteoarthritis [18]. In placebo-controlled double-blind intervention study, a reduced frequency and intensity of attacks in patients with primary Raynaud’s phenomenon after 3 weeks of low-level laser therapy (LLLT) has been demonstrated; however, the mechanisms of these effects remain unclear [16]. Moreover, a significant improvement of LLL treatment in primary and secondary RP patients after 6 weeks and 3 months was noticed [17]. The results of clinical effectiveness of laser therapy reported in mentioned studies are difficult to compare with our findings because of using various application methods and different therapy regiments with different doses, power and wavelengths.

It has been postulated that laser therapy might improve the endothelial function in patients with RP [17]. Although the effectiveness of laser therapy was clinically and thermographically demonstrated in RP patients, no studies analyzing the influence of the MLS treatment on the microvascular abnormalities in NVC exist [16, 17].

In the present study, NVC was used as a non-invasive method for investigating the microvascular abnormalities in RP patients [31]. In this study, 55.3 % of patients with primary RP showed a normal capillaroscopic pattern, and in 44.7 % of cases, minor vascular abnormalities were found. In the group with secondary RP, major capillary abnormalities were observed in 57.5 % patients, moderate changes in 37.5 % and 5 % persons had minor abnormalities. These results are in agreement with other reports, indicating significant differences between the capillaroscopic patterns in patients with primary and secondary RP [9, 29].

A broad spectrum of capillaroscopic findings associated with RP ranging from a normal pattern to microvascular abnormalities and the lack of guidelines for the ill-defined normal range of capillaroscopic changes are the limiting factors for the precise interpretation of capillaroscopic patterns [9]. On the other hand, in the literature, a good reproducibility of the qualitative evaluation was found [30]. Furthermore, the importance of capillaroscopy for identifying RP patients at high risk for the development of scleroderma spectrum diseases was demonstrated [10]. Additionally, a prognostic screening model for RP, which could help to stratify the risk of transition to SSc and to plan an appropriate clinical strategy, was proposed [28].

In our study, after MLS laser therapy in 18.4 % of RP patients with mild changes in NVC, an increase of morphologically unchanged, regular distributed loops was found. Moreover, in seven from 15 patients with moderate changes, an increase of capillaroscopic images with regular distribution and normal capillary density was observed. Furthermore, after MLS laser therapy in 14 of 40 (35 %) patients with secondary RP, an increase in the loop number and a reduction in avascular areas, capillary alterations and microhaemorrhages in NVC were demonstrated.

These findings may confirm the observations of other authors who reported that the endothelium-mediated dilatation of the brachial artery in patients with primary RP can be improved by laser therapy [17]. Although LLLT appears to be an effective treatment in RP patients, different therapeutic effects based on the heterogeneity of the clinical presentation of primary RP have been suggested [16].

The positive effect of MLS therapy on NVC pattern is possible, taking into consideration the results of several findings, suggesting a modulating effect of bosentan [34] or immunosuppressive therapy on scleroderma NVC pattern [27, 35].

In the present study, we demonstrated a significant clinical improvement of MLS laser therapy in patients with primary and secondary RP. According to Gladue, self-reported assessment of RP severity is associated with the possibility of placebo responses [36]. However, a combination of outcome measures could diminish the placebo response. In this study, the clinical assessment included the number of attacks, the mean duration of Raynaud’s attack and severity of pain, according to the visual analogue scale. Moreover, after the MLS laser treatment, the development of nailfold microvascularization, characterized by an increase in the loop number with regular distribution and normal capillary density and a reduction in capillary alterations and microhaemorrhages in NVC, were observed in 35 % of patients with secondary RP.

Recently, many studies have concentrated on the role of angiogenesis and microvascular endothelial injury in the pathogenesis of RP [2]. Dysfunction of microcirculation in the course of RP is associated with homeostasis disturbances between pro- and anti-angiogenic factors, resulting in the impaired vasodilation and the initiation of angiogenesis [2]. Increased production of VEGF and Ang-2 has been demonstrated in various conditions in which vascular pathology is related to endothelial cell activation [37]. In our previous study, we demonstrated a relationship between capillaroscopic abnormalities, serum VEGF concentrations and other endothelial cell activation markers and clinical manifestation in SLE patients [10, 38].

Now, we demonstrated that the VEGF serum concentration was significantly higher in patients with primary and secondary RP compared to healthy volunteers. Moreover, the mean serum concentration of VEGF did not differ significantly between patients with primary and secondary RP before and after MLS laser therapy. In our previous study, we demonstrated significantly higher serum levels of VEGF in SLE patients with severe and moderate microvascular changes compared with patients with mild capillaroscopic abnormalities [11]. Although the results of this study in RP patients confirm that in patients with severe and moderate changes, the serum concentration of VEGF is higher than in patients with mild microvascular abnormalities; no significant differences were demonstrated.

Ang-2 and VEGF coordinately regulate endothelial behaviour. In the presence of VEGF, Ang-2 enables migration and proliferation of endothelial cells and the sprouting of new blood vessels, whereas the presence of Ang-2 leads to the endothelial cell death and vessel regression if the activity of VEGF is inhibited [3]. In previous reports, Ang-2 is shown to be a key serum marker reflecting the activity and severity of vascular diseases [4].

In the present study, the Ang-2 serum concentration was higher in patients with primary and secondary RP compared with healthy volunteers. Moreover, after the MLS laser therapy, a significant decrease of the mean Ang-2 serum levels in patients with primary and secondary RP was demonstrated. Several studies have shown increased Ang-2 concentrations in vascular diseases, including systemic sclerosis (SSc), [39] compared to healthy volunteers. It is postulated that serum level of Ang-2 is a useful marker to evaluate the activity and severity of systemic sclerosis [39]. Furthermore, clinical studies have shown that vasculopathy associated with the elevation of serum Ang-2 levels reflects the inflammatory process underlying high SSc activity [39]. The results of this study confirm the relation between the Ang-2 serum levels and the clinical and microvascular findings after MLS laser therapy in RP patients.

In this study, we investigated the influence of MLS laser therapy on clinical features, microvascular changes in nailfold videocapillaroscopy and circulating modulators releasing as a consequence of vascular endothelium injury such as VEGF and Ang-2 in patients with primary and secondary Raynaud’s phenomenon.

Our results suggest that MLS laser therapy showed beneficial clinical effects manifested by a decrease of duration and number of RP attacks as well as a degree of pain score in VAS scale after 3 weeks of therapy in patients with primary and secondary RP. Furthermore, the correlation between the number of RP attacks and intensity of pain with the microvascular changes in NVC observed in this study may confirm the association between severity of the capillaroscopic abnormalities and progression of the disease. Moreover, the tendency of normalization of Ang-2 concentration in the serum of primary and secondary RP patients may suggest favourable effect of MLS therapy on regulation of processes involved in microvascular disorders.

Our data suggest that MLS laser therapy shows beneficial clinical effects in patients with primary and secondary RP. Furthermore, NVC may reflect microvascular abnormalities associated with clinical improvement after MLS laser therapy RP patients. Moreover, Ang-2 serum level may be a useful marker of microvascular abnormalities in RP patients treated with MLS therapy.

## Abbreviations

RP: Raynaud’s phenomenon
MLS: Multiwave Locked System
NVC: Nailfold videocapillaroscopy
VEGF: Vascular endothelial growth factor and Ang-2, angiopoietin-2
